# Second-opinion stress tele-echocardiography for the Adonhers (Aged donor heart rescue by stress echo) project

**DOI:** 10.1186/1476-7120-8-20

**Published:** 2010-06-01

**Authors:** Daniele Franchi, Davide Cini, Giorgio Arpesella, Sonia Gherardi, Italo Calamai, Giuseppe Barletta, Serafina Valente, Emilio Pasanisi, Stefania Sansoni, Caterina Ricci, Walter Serra, Eugenio Picano, Tonino Bombardini

**Affiliations:** 1 Department of Echocardiography and Medical Informatics, Institute of Clinical Physiology, National Research Council , Via Moruzzi 1, Pisa (56124), Italy; 2 Department of Surgery and Transplants, University of Bologna, Policlinico S. Orsola/Malpighi, Via Massarenti 9, Bologna (40138), Italy; 3 Department of Cardiology, M. Bufalini Hospital, Viale Ghirotti, 286, Cesena (47521), Italy; 4 Intensive Care Department, Usl11 Empoli Hospital, Viale Boccaccio 12, Empoli (50053), Italy; 5 Department of Cardiology, Careggi Hospital, Via Delle Oblate 1, Firenze (50134), Italy; 6 Department of Cardiology, Baggiovara Hospital, Via Giardini 1355, Baggiovara (41100), Italy; 7 Department of Cardiology, Azienda Ospedaliero-Universitaria di Parma, Via Gramsci 14, Parma (43100), Italy

## Abstract

**Background:**

To resolve the current shortage of donor hearts, we established the Adonhers protocol. An upward shift of the donor age cut-off limit (from the present 55 to 65 years) is acceptable if a stress echo screening on the candidate donor heart is normal. This study aimed to verify feasibility of a "second opinion" of digitally transferred images of stress echo results to minimize technical variability in selection of aged donor hearts for heart transplant.

**Methods:**

The informatics infrastructure was created for a core lab reading with a second opinion from the Pisa stress echo lab. To test the system, simulation standard stress echo cineloops were sent digitally from 5 peripheral labs to the central core lab.

Starting January 2009, real marginal donor stress echos were sent via internet to the central core echo lab, Pisa, for a second opinion before heart transplant.

**Results:**

In the simulation protocol, 30 dipyridamole stress echocardiograms were sent from the five peripheral echo labs to the central core lab in Pisa. Both the echo images and reports were correctly uploaded in the web system and sent to the core echo lab; the second opinion evaluation was obtained in all cases (100% feasibility). In the transplant protocol, eight donor cases were sent to the Pisa core lab for the second opinion protocol, and six of them were transplanted in marginal recipients.

**Conclusions:**

Second-Opinion Stress Tele-Echocardiography can effectively be performed in a network aimed to safely expand the heart donor pool for heart transplant.

## Introduction

Donor shortage is a limiting factor in heart transplantation. For instance, 300 heart transplants are performed each year in Italy but there are 800 patients on the heart transplant list. An effective way to solve the current shortage would be to accept an upward shift of the donor age cut-off limit (from the current 55 to 65 years). Of a total of about 1200/year donor pool, 600 donors are aged < 55 years, and 300 of them are eligible for heart donation; since 600 potential donors are aged > 55 years, the recruitment of even one-fourth of the currently dismissed aged donor pool would thereby dramatically decrease the current donor supply shortage [1,2]. Age-related high prevalence of asymptomatic coronary artery disease and cardiomyopathy severely limit the feasibility of this approach, unless a functional screening on the candidate donor heart is performed [3,4]. Pharmacological stress echo is inexpensive, non-invasive and allows a simultaneous evaluation of inducible ischemia and contractile reserve of the left ventricle - therefore, it is capable of unmasking prognostically meaningful occult coronary artery disease or cardiomyopathy [5].

A critical factor in stress echo is the operator's experience. Inter-observer variability can be effectively deflated by some simple criteria, as previously proven by a decade of large-scale international multicenter studies, explicit conservative reading criteria, preliminary quality control of readers, and previous exposure to joint reading sessions [6-8]. In addition, a "second opinion" of digitally transferred images of stress echo results could solve the technical variability in the selection of aged donor hearts for heart transplantation [9,10].

The aim of this study was to verify the feasibility of a "second opinion" of digitally transferred images of stress echo results in selection of aged donor hearts for heart transplantation.

## Methods

### Telemedicine network flow chart

When the Transplant Coordination Center identifies a marginal aged donor, the cardiologist where the donation takes place is alerted, performs resting echocardiography and when it is normal, stress echo [1]. At the end of the stress echo, the cardiologist of the recruiting center selects and stores four cineloops (stress echo quad screen) on any storage media (CD, memory pen) and transfers cineloops on a standard computer connected to the Internet [11]. The cardiologist logs into the transplant website [12]; he transfers the echo cine loops (stress echo quad screen, 'Additional file 1: Movie 1') to the website and on the website fills in resting echo and stress echo forms ('Additional file 2: Movie 2'); after completion of peripheral operations, the website transfers reports and images to the central server in Pisa, and automatically alerts the Pisa cardiologist by sms and e-mail. Once alerted, the core echo lab cardiologist in Pisa opens the website, reads peripheral echo lab reports and views resting and stress echo cine loops.

**Figure 1:**
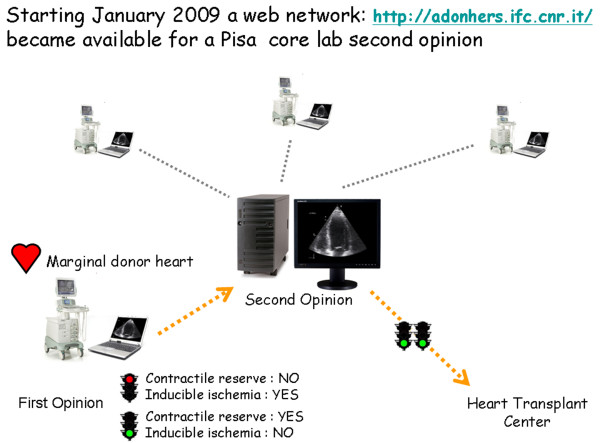
**Telemedicine network architecture with all involved centers**. The accredited cardiologist of the area where the donation takes place, will send, via internet stress echo digitized images to the central core Echo Lab, Pisa (IFC, CNR). Digitized eight-frame cine-loop quad screen echocardiograms are transmitted to the central core Echo Lab laptop computer-receiving station. One core-lab cardiologist is available by telephone or e-mail during the procedure. The Echo Lab is responsible for the "second opinion" of digitally transferred images. One of three cardiologists will interpret the tele-echocardiograms and will make "second-opinion" decisions, and is responsible for the final ("go-not go") green light to donation. When done, the second opinion report is finally sent to the transplant coordination center, and if the marginal heart is eligible for transplant, is proposed to the cardiac surgeon for transplant.

The Pisa cardiologist will interpret the tele-echocardiograms and will make "second-opinion" decisions, with a final ("go-not go") green light to donation [12]. When completed, the second opinion report is finally sent to the transplant coordination center, and if the marginal heart is deemed eligible for transplant, it is proposed to the cardiac surgeon for transplant (Fig. 1).

### Telemedicine network architecture

A computerized web-based system has been developed for automatic data and image transfer and for analysis and report phases. The computer clients used by both the recruiting centers (laptop) and the second opinion center (desktop) connect to the server merely using the ordinary web browser, with no specific software required. A centralized server collects and stores all medical (cineloops, reports, results) and informative (users, procedures) data on a database. The server is located at a second opinion center and is accessed by secure protocol https. All the computer clients used by both the recruiting centers (laptop) and the second opinion center (desktop) connect to the server merely by using a common web browser, without needing to install any specific software [13-15].

#### Communication phases

**Figure 2:**
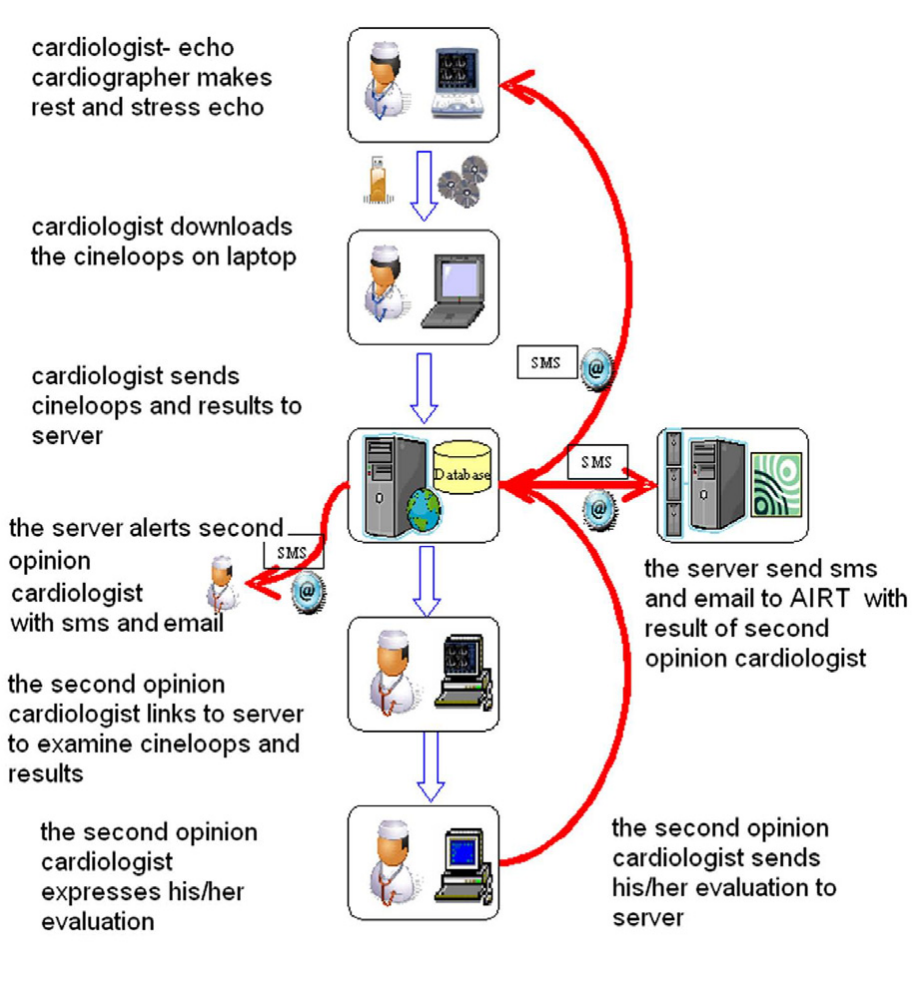
**Phase diagram of the entire communication procedure**. When the Transplant Coordination Center identifies a marginal aged Donor, the cardiologist where the donation takes place is alerted, performs resting echocardiography and when normal, stress echo. At the end of the stress echo the cardiologist of the recruiting center fills out a standard stress echo report; after that he/she selects and stores 4 cineloops (resting and stress echo) on any storage media (CD, memory pen) and transfers cineloops to a standard computer connected with the Internet. By the computer he/she logs into the transplant website and fills out resting echo and stress echo forms on the website. After completing all operations the cardiologist clicks the end of operations. The web site automatically transfers reports and images to the central server in Pisa and automatically the cardiologist of the central echo lab in Pisa is alerted by sms and e mail. Once alerted, the Pisa cardiologist opens the website, reads peripheral echo lab reports and views resting and stress echo cine loops. The Pisa cardiologist will interpret the tele-echocardiograms and will make "second-opinion" decisions and final ("go - not go") green light to donation.

**Figure 3:**
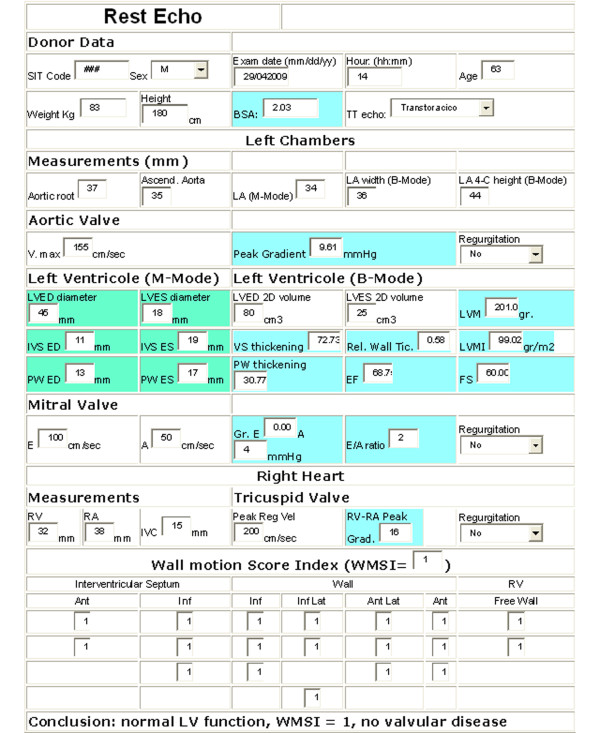
**Form for resting echo report**. Potential donors are recruited for the stress echo protocol if in resting echocardiogram: wall motion score index is completely normal (WMSI = 1), left ventricular ejection fraction > 45%, no signs of diastolic ventricular dysfunction , no significant valve disease , and left ventricular hypertrophy ≤ than mild.

**Figure 4:**
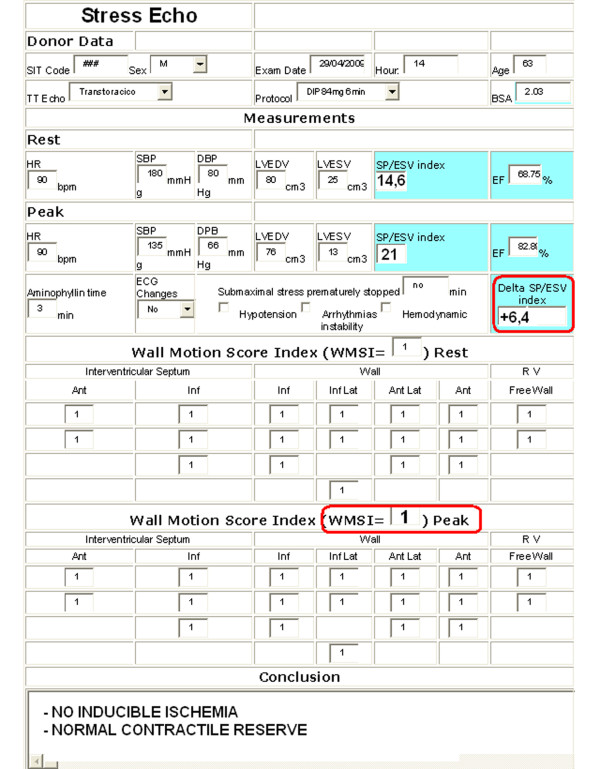
**Form for stress echo report**. The dipyridamole (0.84 mg/kg in 6 min ) "fast" pharmacological stress echo test is performed following the European Association of Echocardiography protocol. The Wall Motion Score Index is calculated in each potential donor at baseline and peak stress, from 1 = normal to 4 = dyskinetic, in a 17-segment model of the left ventricle. A test result is considered positive when the wall motion score increases by one grade or more at peak stress, with at least one normal segment becoming hypokinetic, akinetic or dyskinetic; it had been agreed *a priori *to consider mild hypokinesia. Regional wall motion abnormalities (WMSI > 1.0) exclude the heart from eligibility for donorship, and the phrase "inducible ischemia " automatically appears in the web stress echo report. Once the web form with rest and peak stress LV volume (EDV and ESV) values and pressures is filled out, contractile reserve is automatically calculated by the web system as the SP/ESV (Systolic Pressure/End-Systolic Volume) index increase (from baseline to peak stress). The contractile reserve is automatically reported normal up-sloping when peak exercise SP/ESV index is higher than baseline; abnormal negative, when peak exercise systolic pressure/end systolic volume index is lower than baseline. Donor hearts with abnormal negative contractile reserve are also excluded from donorship even if no signs of inducible ischemia are reported. Prematurely halted submaximal stress are considered non-diagnostic and exclude the heart from donorship.

**Figure 5:**
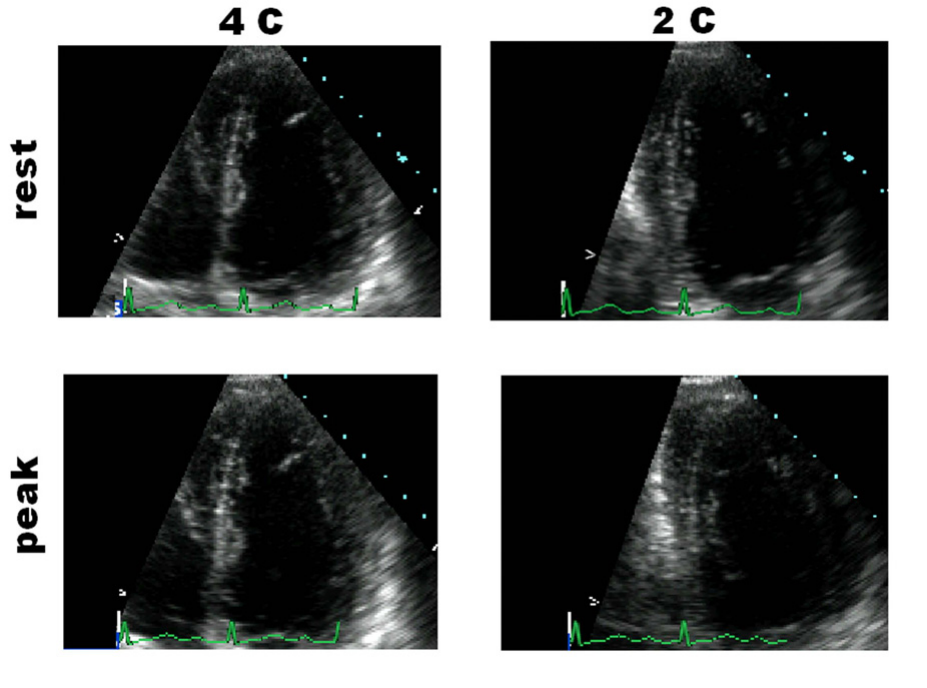
**Rest and stress cineloops stored on the server**. *Normal stress echo of a marginal donor*. When the Network for Organ Sharing identifies a marginal aged donor, the local cardiologist is alerted; he performs rest (upper panels) and stress-echo (lower panels, peak stress). At the end of the procedure the cardiologist selects and transfers the four cineloops to the core lab. In this example of normal stress echo of a marginal donor, the wall motion was normal at baseline and at peak stress (WMSI = 1 at baseline and peak stress), without signs of stress-inducible ischemia. The pressure/volume relation was 8 mmHg/ml/m^2 ^at baseline, increasing to 14 mmHg/ml/m^2 ^at peak stress, demonstrating the absence of latent myocardial dysfunction. Based on the dipyridamole stress results, despite an age beyond the 55-year limit, the heart was chosen for orthotopic heart transplantation, and was explanted using standard technique.

The sequence of the entire communication procedure is shown in the phase diagram of the operation in Fig. 2. Before starting to operate the first time, each user (the cardiologists of both the recruiting and the second opinion centers) must be registered by the server administrator who assigns him/her an account and an exclusive memory area on the database server. Only the cardiologist user (and the second opinion cardiologist) might then access his/her data in protected and reserved mode. The first web page allows the transfer of the four cineloops; the next web pages permit the cardiologist to fill in the resting (Fig. 3) and stress echo (Fig. 4) medical report in standard mode. Some measures and indexes are obtained automatically by software processing. When both forms are filled in and stored on the database, the second opinion request is started by the server sending an sms and an email. The echo lab in Pisa is responsible for "second opinion" on digitally transferred images. One of three cardiologists will interpret the tele-echocardiograms and will make "second-opinion" decisions (Fig. 5 and 'Additional file 1: Movie 1'). Controls and timers are scheduled for monitoring the correct procedure to store reports and cineloops and to await the second opinion answer (timeout expired aborts the request). The results of the first and second opinion are sent to the coordinating center of AIRT (Associazione Interregionale Trapianti).

### Simulation Protocol

To test the function of the Second-Opinion Stress Tele-Echocardiography system, simulation cases (i.e. anonymous standard dipyridamole stress echo reports and quad screen cineloops in in-hospital patients) were digitally sent from five peripheral echo labs to the central core lab. The ability of peripheral cardiologists to upload reports and cineloops in the web system was evaluated. The readability of images in the central echo lab was evaluated.

### Transplant protocol

Starting January 1, 2009, the informatics infrastructure [12] ('Additional file 2: Movie 2') became available for a core lab reading with a second opinion from the central Pisa stress echo lab. Before every center begins to recruit possible donors, the cardiologist who will perform and evaluate stress echo has to pass strict quality control testing for reading stress echo [16]. Once certified, the cardiologist is entitled to enroll patients. The second reader was a cardiologist highly experienced in stress echocardiography and only a unanimous negativity decision was accepted. In the case of a split decision (positivity vs negativity) of the two readers (peripheral and central), the heart was not considered eligible for donation.

### The dipyridamole stress echo protocol

**Figure 6:**
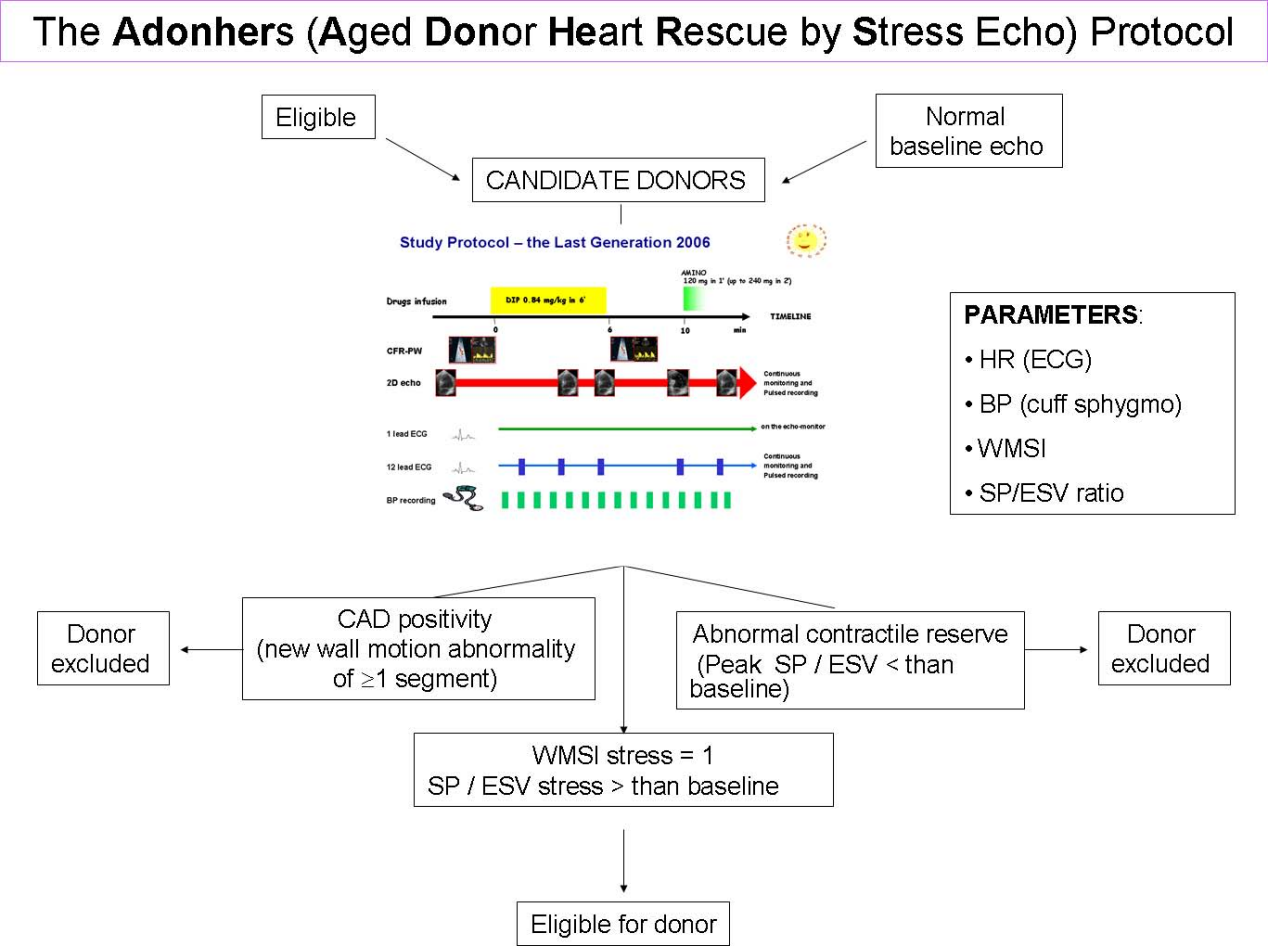
**The dipyridamole stress echo in the Adonhers protocol**. When resting echocardiography was normal a pharmacological stress echo test was performed using dipyridamole (0.84 mg/kg in 6 min ). We accepted a priori three criteria of stress echo positivity, excluding the heart from eligibility for donorship: 1) Regional wall motion abnormalities at rest or during stress. 2) A LV elastance falling during stress. 3) A submaximal stress halted due to non-diagnostic limiting effects before completion of the infusion, since a submaximal test dramatically lessens diagnostic and prognostic power. Accepting a heart was done in conformity with clinical and emergency criteria in use.

When resting echocardiography was normal a pharmacological stress echo test was performed following the European Association of Echocardiography (EAE) protocol [7], using dipyridamole (0.84 mg/kg in 6 min) (Fig. 6). Where there were contraindications to dipyridamole (asthma, hypotension, bradyarrhythmias), the second-choice drug was dobutamine (up to 40 mcg/kg/min). The echo images were tape-recorded and periodically digitized. During the procedure, pressure and ECG were recorded every minute. Brachial blood pressure was measured with cuff sphygmomanometer. In each phase of the stress echo, the projections of the four chambers and of the apical two chambers were recorded to obtain the left ventricular end-systolic volume by biplane Simpson rule in order to calculate the left ventricular (LV) elastance (systolic pressure/left ventricle end-systolic volume ratio) [17-20]. The diagnostic end points were: the development of obvious echocardiography positivity, obvious alterations of ECG (ST segment shift > 3 mm). The test was halted in the case of hypotension (relative or absolute) with decrease in blood pressure > 30 mmHg. A non-maximal diagnostic stress excluded donation since it provides inadequate diagnostic and prognostic information. Regional wall motion score index was assessed and graded on a scale from 1 (normal) to 4 (dyskinetic) at rest and after stress in each of the 17 segments [7]. Left ventricular wall motion core index was calculated by summing the scores and dividing the sum by 17. By definition, donors with abnormal stress echocardiography had rest wall motion abnormalities and/or stress-induced wall motion abnormalities. We also considered the changes in left ventricular volumes as an index of global dysfunction [19] and pressure/volume changes as an index of LV elastance [17,18]. The echocardiographic study is divided into three different parts:

1 - Segmental wall motion: the essential step for the evaluation. The donors with abnormal wall motion at baseline or during stress will be excluded from donorship.

2 - End-systolic pressure-volume determination (the contractile reserve). The LV force is determined at rest and at peak stress as the ratio of the systolic pressure (cuff sphygmomanometer)/end-systolic volume index (biplane Simpson rule/body surface area). The contractile reserve is automatically calculated by the web system as the SP/ESV (Systolic Pressure/End-Systolic Volume) index increase (from baseline to peak stress). The contractile reserve is normal up-sloping when peak exercise SP/ESV index is higher than baseline; negative, when peak exercise systolic pressure/end systolic volume index is lower than baseline. Donor hearts with abnormal negative contractile reserve are excluded from donorship.

3 - The measure of coronary flow reserve (CFR): an important parameter, but not essential for the study. It is necessary to obtain a pulsed flowmetry Doppler of the anterior descending coronary artery at baseline and during stress [21]. The evaluation is not completed in a percentage of donors (around 40%). However, the lack of information about pulsed Doppler flowmetry is not mandatory for the screening procedure.

Prematurely halted submaximal stress (e.g., hemodynamic instability) loses diagnostic and prognostic power, and is unacceptable in the transplant setting [6,7].

### Statistical Analysis

Agreement between stress tele-echocardiography and conventional echocardiographic interpretation was evaluated with the kappa statistic (expressed with 95% confidence intervals). Overall accuracy (defined as the number of correct clinical diagnoses divided by the number of diagnoses) was compared with the McNemar test to determine whether conventional and tele-echocardiographic interpretations differed significantly. Mean values are reported as mean ± SD. All calculations utilized software SPSS 16.

## Results

### The informatics infrastructure

The informatics infrastructure is available on the web, linking to http://adonhers.ifc.cnr.it[12] (a video graphically explains the procedure, 'Additional file 2: Movie 2'). A network of recognized cardiologists with echocardiographic skills (American Society of Echo level III) able to properly execute and interpret stress echo studies bedside, were accredited in each center. The central core echo lab in Pisa (IFC, CNR) was responsible for identification and certification of a stress echocardiographer in each center; "second opinion" of digitally transferred images.

### Simulation protocol

Simulation cases were digitally sent from five peripheral echo labs to the central core lab in Pisa. Each peripheral echo lab uploaded and sent six simulation cases (three with normal and three with abnormal stress echo response). Both the echo images (stress echo quad screen cine loops) and reports were correctly uploaded in the web system and sent to the core echo lab. Images were readable and the second opinion evaluation was feasible in all cases (100% feasibility). Second opinion evaluation concordance with the peripheral lab was present in 28/30 cases and discordance in 2/30 cases (Table 1).

**Table 1:** Simulation protocol results

Peripheral center	Correctly uploaded stress echo cineloops	Correctly filled rest and stress reports	Digitized transmission to Pisa	Images readability in Pisa	Second opinion discordance for WMSI	Second opinion discordance for contractile reserve
Center #1	6/6	6/6	6/6	6/6	-	-

Center #2	6/6	6/6	6/6	6/6	1	-

Center #3	6/6	6/6	6/6	6/6	-	-

Center #4	6/6	6/6	6/6	6/6	1	-

Center #5	6/6	6/6	6/6	6/6	-	-

### Transplant protocol

**Figure 7:**
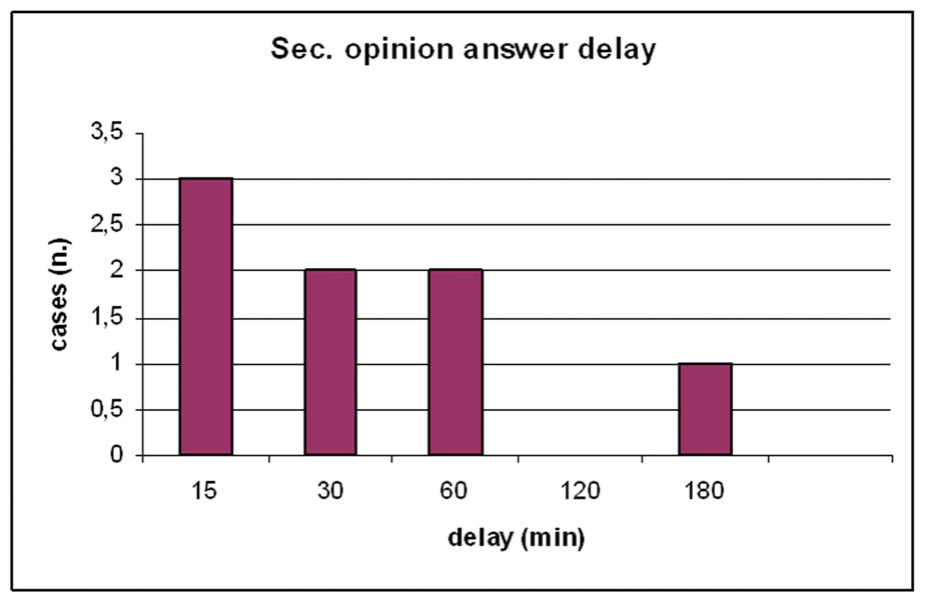
**Second opinion cardiologist answer delay for the 8 marginal Donor hearts**. Second opinion answer delay ranged from 15 to 180 min. In all cases the second opinion was largely sent before the end of the observational period and organ harvesting by the cardiac surgeon occurred after completion of the stress echo evaluation.

Starting January 1, 2009 the informatics infrastructure [12] became available for a core lab reading with a second opinion from the central Pisa stress echo lab. Eight donor stress echo cases were sent to Pisa for the second opinion protocol. Image and report charge from the peripheral centers, transmission to Pisa and second opinion protocol were feasible in all cases (100% feasibility). Second opinion answer delay is displayed in Fig. 7. Agreement between conventional and tele-echocardiographic interpretations was present in all cases. A final clinical diagnosis was obtained from cardiac catheterization findings in the transplanted heart and by anatomic study in non-transplanted hearts [2].

## Discussion

Age restrictions (< 55 years) in cardiac donors are being relaxed due to limited donor supply.

To resolve the current shortage of donor hearts we started the Adonhers (Aged Donor Heart Rescue by Stress echo) Project in which an upward shift of the donor age cut-off limit (from present 55 to 65 years) was accepted [1]. Due to the age-related high prevalence of asymptomatic coronary artery disease and cardiomyopathy, a stress echo screening on the candidate donor heart was scheduled in the protocol [20]. Cardiologists who are competent and skilled in stress echocardiography can perform dipyridamole stress [6,7]. They must be familiar with digitizing echocardiograms and transmission [13,16,22]. Most important, cardiologists must be willing to respond to emergency calls [9]. Training, coordination and support by cardiologists and emergency physicians are essential [23]. A background in stress echocardiography entitles the cardiologist to interpret dipyridamole stress echo results [16].

Rapid interpretation of resting echocardiograms and ECGs allowed stress testing to be performed within 3 h of brain death. Essential features of the proposed system is the ease of use, the efficiency of the database and the effective alerts to the second opinion cardiologist and to the AIRT center. Minor problems to transmission occurred in only two cases: in one event the echocardiography CD burner was not working, making transmission impossible, and in the other event the cineloops were sent slowly due to the large amount of data on file (greater than 25 MB).

When a stress echo is scheduled in the Adonhers protocol, interest is focused on wall motion segmental contraction abnormality to diagnose ischemic response to stress [1,2,20] and on systolic pressure/end-systolic ratio to assess contractile reserve; the critical level to define the presence of contractile reserve is defined as an increase of at least 5% (in absolute terms) and it is easily calculated by the web system. A negative contractile response may reflect a global ischemic and/or catecholamine burden, a decrease in the number of fully functional myocytes, a decrement in myocyte function, or a combination of these mechanisms. In practical terms, this implies that a stress echo test with hemodynamic instability or lack of global hyperkinetic response and left ventricular volume reduction should be dismissed even in the absence of regional wall motion abnormalities.

### Vasodilator vs. inotropic stress testing in the Adonhers protocol

Dobutamine, and vasodilators (at appropriately high doses) are equally potent ischemic stressors for inducing wall abnormalities in the presence of a critical epicardial coronary artery stenosis [5]. Dipyridamole acts through reduced subendocardial flow supply subsequent to inappropriate arteriolar vasodilation and steal phenomena and Dobutamine through catecholamine induced increased myocardial oxygen demand [7]. However a inotropic stress is potentially harmful in the particular setting of heart donation. The adrenergic storm,. in the phase preceding brain death, is associated with a prolonged release of norepinephrine from cardiac sympathetic nerve endings and leading to direct myocardial injury and/or coronary vasospasm [2]. The secretion of large amounts of endogenous catecholamine, as may occur in subjects with subarachnoid hemorrhage, has been associated with the appearance of transient left ventricular apical diskinesis [3,4]. Catecholamine may exert a direct toxic effect on the myocardium through changes in autonomic tone, enhanced lipid mobility, calcium overload, free radical production, or increased sarcolemmal permeability. Further more all donors are managed according to standardized organizational guidelines [1] that included: the use and inotropic agents (preferably noradrenalin or dopamine) to maintain a systolic blood pressure (SBP) > 90 mmHg, central venous pressure (CVP) 4 to 12 mmHg and urine output 1 to 2 ml/kg/h. A dobutamine instead of dipyridamole stress echocardiography may induce or enhance the typical myocardial catecholamine necrosis frequently observed at histological studies of brain dead heart donors [2].

### The "second opinion" telemedicine

Evolving telemedicine technology has the potential to improve access to echocardiography diagnoses in the intensive care unit and emergency room [9,10]. The two primary modes of telemedicine practice are "store and forward" and "real-time" videoconferencing [13]. A digital echocardiogram (often several cardiac cycle loops) can be stored at one site and forwarded across a telemedicine network to a receiving station for review at a later time and "second opinion" procedure. Previous studies demonstrated that echocardiographic telemedicine (*tele-echocardiography*) for emergencies can provide rapid, 24-h consultation [24] and that dobutamine stress tele-echocardiography in the emergency department is feasible [25]. Normal results on stress echocardiography in the emergency department may obviate the need for hospital admission in patients presenting with noninfarction chest pain. Implementation of this program appears to be practical in the clinical setting but requires cooperation and commitment on the part of emergency physicians, cardiologists, nurses and sonographers. Use of modern communication technology in this context, is the sole decisive factor that makes such telemedicine system successful. Early results in brain dead marginal heart donors showed that the system is reliable, functions with a clinically acceptable performance, and transfers medical data with a reasonable quality, Thus, the system is applicable, and might be generalized in clinical practice in cardiology.

## Conclusions

Second-opinion stress tele-echocardiography for aged donor heart selection can safely and effectively be performed in candidate heart donors with brain death, and shows potential to extend donor criteria in heart transplantation. This model of telemedicine technology tested in the extreme clinical setting of brain-dead heart marginal donors could be expanded to other critical clinical settings.

## Competing interests

*Funding Sources*

Partial funding for this project was provided by Heart and Lung Transplantation Program, Regione Emilia-Romagna (1ASTEARP).

## Authors' contributions

DF, DC participated in the design of the study, developed hw/sw system and web design and helped to draft the manuscript; T.B. conceived this study, performed the data analysis, and drafted the manuscript; GA, SG, IC, GB, SF, EP, SS, CR, and WS were responsible for data collection and revised the manuscript; E.Pi. gave a contribution to preparation of study design, data discussion, and critical revision of the manuscript. All authors read and approved the final manuscript.

## Supplementary Material

Additional file 1 **Sample of 4 echo stress cineloops**. stress echo cineloops. Sample of rest and stress cineloops sent and stored on the server Click here for file

Additional file 2 **Second-Opinion Stress Tele-Echocardiography project presentation**. Adonhers movie. Short movie of Second-Opinion Stress Tele-Echocardiography project presentation When the Network for Organ Sharing identifies a marginal aged Donor, the local cardiologist is alerted, performs rest and when normal, stress-echo. At the end of the procedure the cardiologist selects and transfers 4 cineloops to the Core -Lab. The Pisa cardiologist is responsible for "second-opinion" decisions and final ("go - not go") green light to donation. If eligible, the heart is proposed to the surgeon for transplant. Click here for file
